# Acupuncture to Treat Sleep Disorders in Postmenopausal Women: A Systematic Review

**DOI:** 10.1155/2015/563236

**Published:** 2015-08-23

**Authors:** A. G. Bezerra, G. N. Pires, M. L. Andersen, S. Tufik, H. Hachul

**Affiliations:** ^1^Departamento de Psicobiologia, Universidade Federal de São Paulo, Rua Napoleão de Barros 925, Vila Clementino, 04024-002 São Paulo, SP, Brazil; ^2^Departamento de Ginecologia, Universidade Federal de São Paulo, Rua Napoleão de Barros 715, 7° Andar, 04024-002 São Paulo, SP, Brazil; ^3^Departamento de Ginecologia, Casa de Saude Santa Marcelina, Rua Santa Marcelina 177, 08270-070 Itaquera, SP, Brazil

## Abstract

Sleep disorders are commonly observed among postmenopausal women, with negative effects on their quality of life. The search for complementary therapies for sleep disorders during postmenopausal period is of high importance, and acupuncture stands out as an appropriate possibility. The present review intended to systematically evaluate the available literature, compiling studies that have employed acupuncture as treatment to sleep disorders in postmenopausal women. A bibliographic search was performed in PubMed/Medline and Scopus. Articles which had acupuncture as intervention, sleep related measurements as outcomes, and postmenopausal women as target population were included and evaluated according to the Cochrane risk of bias tool and to the STRICTA guidelines. Out of 89 search results, 12 articles composed our final sample. A high heterogeneity was observed among these articles, which prevented us from performing a meta-analysis. Selected articles did not present high risk of bias and had a satisfactory compliance rate with STRICTA guidelines. In general, these studies presented improvements in sleep-related variables. Despite the overall positive effects, acupuncture still cannot be stated as a reliable treatment for sleep-related complaints, not due to inefficacy, but rather limited evidence. Nevertheless, results are promising and new comprehensive and controlled studies in the field are encouraged.

## 1. Introduction

Considering the current increase in life expectancy in the worldwide population [1], it is worth to turn attention to health and quality of life in postmenopausal women [2]. This period is of major importance to women's life, with important repercussions in social and clinical aspects. The great changes that women undergo during this period usually affect women's quality of life negatively [3]. Among them, one may highlight alterations that take place as a consequence of the normal aging process, both clinically and behaviorally, such as increased tendency or prevalence of certain diseases, social isolation, and empty nest syndromes, among others [2]. However, consequences not only related to aging but rather to hypoestrogenism deserve special attention, either due to its burden on public health costs or to women's quality of life. In general, hormonal alterations during menopause transition and postmenopause are strongly related to a series of unpleasant symptoms and complaints [3], among which the vasomotor symptoms, hot flushes, mood disorders, and specially sleep-related disorders [4] are remarkable.

The most prevalent sleep disorder during this phase is insomnia, being characterized by a difficulty in initiating or sustaining sleep [5]. In this case, insomnia may occur either as a condition during menopause transition or as a maintenance or worsening of a previously established condition, with prevalence ranging from 25% to 60% among these women [6, 7]. Some specific characteristics of postmenopausal period may predispose to insomnia, such as the hormonal alterations, the high prevalence of anxiety and mood disorders, and vasomotor symptoms, among others [4]. Another highly prevalent sleep disorder that affects postmenopausal women is the obstructive sleep apnea syndrome (OSAS), notable for its episodes of upper airway obstructions, which ultimately lead to fragmentation of sleep and to a decreased sleep efficiency [8]. It is estimated that more than a half of women in this period presents this disorder. Some intrinsic features of postmenopause may explain such a high prevalence, such as increased body weight and hormonal alterations [9, 10]. In this case, increased body weight contributes to the incidence of OSAS by increasing the airflow resistance and leading to upper airway obstructions. Hormonal alterations lead to OSAS mainly due to the decrease in progesterone levels, which is described as a mediator of the respiratory drive during sleep [11, 12]. Furthermore, all these sleep disorders and complaints lead to important daytime consequences, such as irritability, lack of attention, and anxiety.

The treatments for menopausal symptoms have been discussed for many years, and hormonal therapy is always an important issue, due to its well-known positive effects upon certain complaints, such as on vasomotor symptoms and on vaginal atrophy. However, since the release of Women's Health Initiative (WHI), which has associated hormonal therapy with an increased risk for breast cancer and thromboembolism [13], this treatment has been more cautiously considered, not being anymore prescribed to every woman indiscriminately and in a generalized way. Regarding sleep disorders during menopause transition and postmenopause, most treatments are generically indicated but eventually present diminished efficacy or unwanted side effects. In the cases of insomnia, the most common therapy relies on the use of hypnotic drugs and benzodiazepines, which use is many times controverted due to its side effects (both behavioral and physiological) and its possible potential to elicit dependency and tolerance [14, 15]. For OSAS, the most frequent treatment alternatives are weight loss and the use of intraoral or continuous positive air pressure (CPAP) devices [16]. However, both treatments present their flaws: obesity during menopause may be related to hypothyroidism, which is common among this population, together with the natural slowing on metabolism, which predisposes to OSAS and complicates the process of losing weight, while intraoral devices and CPAP may present a reduced compliance rate [9, 17]. In face of this clinical framework, in which standard treatments are not as efficient as intended, the search for alternative and complementary therapies to assist in the relief or reduction of symptoms becomes important.

Several alternative and complementary therapies have emerged as potential treatments for sleep disorders in postmenopausal women, such as lifestyle alterations and physical exercise, homeopathy, phytotherapy (e.g.,* Valeriana officinalis*), isoflavone, yoga, and massages [18]. Among the available therapies, acupuncture stands out as an interesting alternative. This set of techniques has been frequently used as adjuvant on the treatment of many diseases. Indeed, its use has shown efficacy as an adjuvant in many different clinical conditions [19–21], including on the treatment of sleep disorders [22–24]. However, its clinical potential for the treatment of sleep complaints in postmenopausal women has never been systematically reviewed. Thus, the present report intends to make a comprehensive systematic review of the literature about the effects of acupuncture techniques on the sleep disorders and complaints in postmenopausal women.

## 2. Methods

The systematic review protocol detailed below had the aim of selecting articles evaluating the effect of acupuncture on sleep-related complaints and disorders in postmenopausal women and was conducted in accordance with PRISMA (Preferred Reporting Items for Systematic Reviews and Meta-Analysis) [25, 26].

### 2.1. Bibliographic Search

In order to identify the studies that have addressed the potential therapeutic effects of acupuncture in postmenopausal sleep disorders, a systematic bibliographic search was conducted concurrently in PubMed/Medline and in Scopus. Primary search strategy was the following: [acupuncture AND menopause AND (sleep OR insomnia OR apnea)]. Articles were selected in a two-step process: in the first step, titles and abstracts were screened in accordance with their suitability with the purposes of this review. Second step included full text evaluations for eligibility and data extractions. Secondly, articles that were not found in primary search but that were cited by these studies and that were relevant according to the aims of this research were also considered eligible. Inclusion and exclusion criteria were applied in both steps and two authors (AGB and GNP) performed articles selection concurrently.

### 2.2. Inclusion and Exclusion Criteria

Articles were included or excluded according to the evaluation of five different items: type of article, language, population, acupuncture technique, and sleep evaluation.

#### 2.2.1. Type of Article

Only original, interventional, and longitudinal articles were included; no additional limitations regarding study designs were applied. Thus, since case reports to randomized controlled clinical trials were considered eligible, observational or transversal articles were excluded, as well as reviews and other theoretical articles.

#### 2.2.2. Language

To be included, articles should have presented an abstract in English; articles that failed to present an abstract or that presented it in any other language were excluded during the first phase of our selection process (title and abstract screening). No language restriction was applied in the second phase of our selection process (full texts evaluation).

#### 2.2.3. Population

The current review considered only articles in which population was composed by postmenopausal women. Thus, any study conducted in male individuals or in women in pre- or perimenopausal periods was excluded. Specially, articles conducted in breast cancer patients or survivors, in which menopause may have been induced by chemotherapy and hormonal suppression, were excluded in order to avoid heterogeneity within the sample.

#### 2.2.4. Acupuncture

Only articles that have employed traditional/body acupuncture and/or ear acupuncture/auriculotherapy as intervention were included. Articles in which main intervention was based on correlated techniques, such as moxibustion, scraping therapy, and cupping therapy, were excluded. There were no restrictions regarding treatment duration, therapy session duration, or number of sessions until outcome assessment.

#### 2.2.5. Sleep Assessment

Controlled trials presenting parallel intervention and control groups were included. Additional study designs (case reports and cohorts) presenting sleep evaluations in at least two time points, one before and another after acupuncture interventions, were also included. No restriction was applied regarding the type of sleep evaluation, being eligible both objective measurements (polysomnography, actigraphy, etc.) and subjective scales (questionnaires, indexes, scores, etc.).

### 2.3. Risk of Bias Assessment

Potential methodological biases affecting the selected articles were evaluated using the Cochrane risk of bias tool [27]. This tool was developed by the Cochrane Collaboration in order to standardize the evaluation of risk of bias in clinical trials and currently is the most important tool available for this purpose. This tool allows classifying the impact of each potential source of bias as of low, high, or unclear risk. Seven different items are evaluated through this tool: random sequence generation and allocation concealment (selection bias), blinding of participants and personnel (performance bias), blinding of outcome assessors (detection bias), incomplete outcome data (attrition bias), selective outcome reporting (reporting bias), and other sources of bias. As this tool was developed for the evaluation of bias sources usually faced in clinical trials, we did not apply it in case reports. For other comparative nonrandomized studies (such as cohorts), an adaptation of this tool was used, in which items related to randomization, allocation concealment, and blinding were excluded. Risk of bias assessment was performed by two authors concurrently (AGB and GNP).

### 2.4. Evaluation of Articles According to the STRICTA Reporting Guidelines

The STRICTA (Standards for Reporting Interventions in Clinical Trials of Acupuncture) [28] is a reporting guideline derived from the CONSORT (Consolidated Standards of Reporting Trials) checklist and specifically directed to controlled trials of acupuncture. This guideline has emerged from the need to standardize the reporting of clinical trials that involve acupuncture, especially in what regards the reporting of the employed interventions, improving the methodological quality and easing their interpretation and replication. It consists of six items (encompassing 17 subitems) that ideally should be reported in every acupuncture trial. These items refer to the acupuncture rationale, details of the needling protocol, treatment regimen, other components of treatment, the practitioner/acupuncturist background, and the control interventions.

We used the STRICTA guidelines to assess the methodological completeness of the articles selected in this systematic review. It is worth to mention that these guidelines were not designed to be applied as an evaluative tool of prepublished studies (as are the Cochrane risk of bias tool, the Jadad scale, and others [27]), but rather as a guideline intended for authors and researchers, guiding their trials and mainly the reporting of the methods employed. However, as there is no currently available tool to evaluate the methodological soundness of previously published acupuncture trials, we have adapted the STRICTA guidelines for this purpose. For that, we evaluated the reporting of each of the STRICTA's subitems in all the selected articles. Possible answers for each of the subitems were “yes” (if a subitem was properly reported), “no” (if it was not reported or omitted), partially (it a subitem was partially or marginally reported), or nonapplicable (such as subitems related to control interventions in case reports).

Of note, subitems rated as “no” are not conditionally indicative of low methodological quality or of incompleteness, as some of STRICTA subitems do not refer to mandatory practices in clinical acupuncture trials. As an example, a “no” attribution to subitems such as “response sought” (2d) or “needle stimulation” (2e) does not refer to lack of quality but rather may indicate that these practices were not employed in a given protocol. Thus, the use of STRICTA in the present review and the raised data should be interpreted carefully; overgeneralizations and the use of STRICTA to generate quality indexes should be avoided. The evaluation of selected articles according to the STRICTA guidelines was performed by two authors concurrently (AGB and GNP).

## 3. Results

### 3.1. Selected Studies and Sample Description

**Figure 1:**
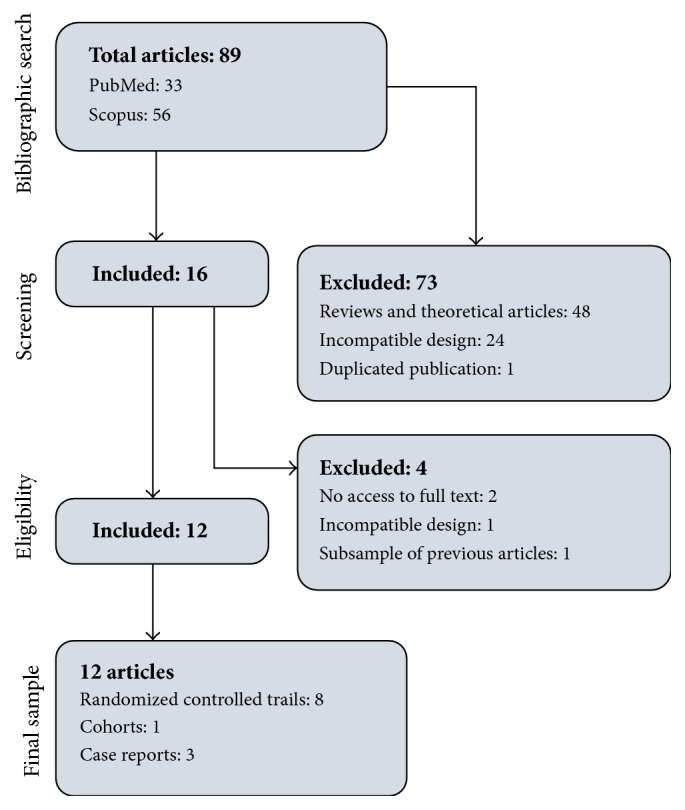
Flowchart of articles selection of the current study.

A total of 89 articles returned as a result of our bibliographic search. Among them, 33 were obtained in PubMed/Medline search and the remaining 56 were found exclusively in Scopus. After title and abstract screening, 73 articles were excluded due to different reasons and 16 articles remained eligible. From these, four more articles were excluded in the second step of the selection process, and the remaining 12 articles composed our final sample, among which 11 were published in English and one in Spanish language [29]. No article was included based on secondary search. The whole selection process is available in Figure 1.

**Figure 2:**
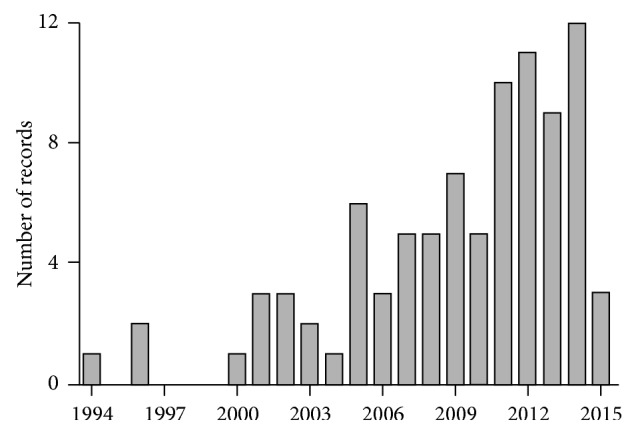
Time analysis of publication on the use of acupuncture for postmenopausal sleep-related complaints.

Most of the screened articles were published in the last two decades, showing that, despite being a millenary technique, reports of the use of acupuncture for postmenopausal sleep-related complaints are very recent. The first report was published in 1994 and since then, a clear temporal growth tendency can be observed. Some few erratic dynamics can be noted, such as the decreases in publication in the years of 2004, 2010, and 2013; however, they can be understood as artifacts of the still reduced total number of articles. As this is a very recent publication field, as well as due to its steady growth, almost 50% of the total output was published in the last five years (Figure 2).

Among the 12 articles that composed our sample, three are case reports and the remaining nine articles are proper comparative studies. A full description of these articles and their main features is available in Table 1. Our primary intention was to perform a meta-analysis based on the results of this systematic review. However, due to the limited number of articles and, more importantly, to the marked methodological heterogeneity among experiments, this was not possible. Therefore, we turned the current report to a narrative review, describing and comparing the selected articles qualitatively, as can be observed in the following sections.

### 3.2. Descriptive Results

Among the 12 selected articles, nine (75%) presented improvements in sleep complaints following acupuncture, while only three (25%) showed nonsignificant effects [30–32]. However, two of the articles presenting positive results have some different characteristics that should be highlighted: Cohen et al. [33] described improvement in self-reported sleep disorders severity when comparing pre- and posttreatment within experimental group; however, nonsignificant effect was observed in between-group comparison. Borud et al. [34] also reported significant results, but in this case authors were measuring long-term effects in a 6–12 months follow-up.

About treatment conditions, a wide range of protocols was used in what regards theoretical background, type of acupuncture intervention, treatment duration, number of sessions, and chosen acupoints. Among the 12 articles, eight have employed exclusively body acupuncture, all of them based on Traditional Chinese Medicine (TCM) background. One article has employed ear acupuncture only, and the remaining two articles employed a mixed protocol, composed of both body and ear acupuncture. Regarding number of sessions, data ranged from nine to 36 sessions, in frequencies ranging from weekly to three times a week. Acupoints ranged enormously among the experiments and no specific pattern or preferable treatment option was observed.

For sleep assessment tools, some degree of variability was also observed. Six experiments (50%) employed self-reported scales related to sleep, which are the most unspecific and least reliable way to measure sleep disorders or related complaints. Sleep logs, which are still a kind of self-report but with a bigger reliability, were employed in one article (8.3%). Then, standardized and validated questionnaires were used in seven articles: the Pittsburgh Sleep Quality Index (PSQI) was used in four articles (25%), Women's Health Questionnaires (WHQ) in two (16.6%), and Women's Health Initiative Insomnia Rating Scale in a single experiment (8.3%). Lastly, polysomnography, which is the gold standard method for sleep evaluation, was used in only one article (8.3%). Of note, only two articles took sleep-related complaints as primary outcomes or aims [35, 36], while the remaining observed sleep as a secondary measure. Moreover, all studies focused only on sleep-related subjective reports and insomnia complaints; no article reported other sleep disorders, such as OSAS.

Regarding article types, we could identify three case reports (25%), one cohort (8.3%) and eight randomized controlled trials (encompassing one multicentric study and a follow-up—66.6%). As these different study designs involve different methodological details and different levels of evidence, results are presented in detail below.

### 3.3. Case Reports

Hu [37] described a case report of a 51-years-old postmenopausal women, in which the treatment was not specifically directed to sleep, but rather to the whole postmenopausal syndrome, which encompassed sleep disorders. The authors used the concepts of postmenopausal syndrome based on TCM diagnosis. In the course of two weeks the authors reported an overall improvement in the symptoms, including the difficulties to sleep, despite no further detail was given. A second report discussed the case of a 49-years-old postmenopausal woman [38]. The patient reported an overall improvement in insomnia complaints, associated with improvements in migraine and vasomotor symptoms, after 22 sessions of acupuncture. The fact that the efficacy of the acupuncture treatment allowed a reduction on the standard pharmacological treatment stands out. The final report addressed the case of a 59-year-old postmenopausal woman who has been complaining about sleep problems for ten years. In this case, ten acupuncture sessions combined with ear acupuncture were sufficient to total symptoms remission [29].

These three reports observed positive effects of acupuncture on sleep-related postmenopausal symptoms. However, the case reports represent the lowest degree of scientific evidence. This type of articles is markedly prone to be affected by publication bias, as negative case reports are completely unlikely to be published. Thus, these cases are useful to disclose the plausibility of the use of acupuncture as an adjuvant on the treatment of the refereed sleep complaints; but the extent of this usefulness and the likelihood to extrapolate these results to a wider sample is questionable. Another caveat relies on the fact that all the reports have employed unreliable sleep measurement tools and failed to provide further details on sleep evaluation. Conversely, as a positive feature of these case reports, this type of article allows an individualized and customized treatment, which is extremely valuable under the concepts of TCM.

### 3.4. Cohort

A single cohort article was included in our systematic review, which encompassed a group of 45 postmenopausal women with insomnia complaints. In this case, the treatment consisted of auricular acupuncture, with acupressure being recommended daily before sleep. Results showed a reduced latency to sleep and an increased total sleep time and sleep efficiency, measured by means of the PSQI [36].

### 3.5. Randomized Controlled Trials

Out of the 12 selected articles, eight were categorized as randomized controlled trials [30–35, 39, 40], corresponding to two-thirds of our sample. Among these, only one article had the treatment of sleep disorders as primary aim or outcome [35], while in the other articles sleep was measured secondarily or indirectly. Total treatment time and treatment frequency varied among studies (nine to 36 acupuncture sessions), as well as the chosen acupoints. In this sense, a single article employed an individualized and customized treatment, in which the acupuncturists were free to choose the treatment in a case by case basis, adapting it to each patient [40].

Regarding the main results of the randomized controlled trials, five of them presented improvements in sleep-related complaints after acupuncture, one of them addressing it in a 6–12-month follow-up. Additionally, one of these articles presented improvement only in within-group analysis, when comparing pre- and posttreatment values within the experimental group, but failed to show significant results in between-group comparisons. Other three articles failed to show significant results of acupuncture on sleep complaints.

Randomized controlled trials are among the methodological designs that provide the greatest levels of scientific evidence, usually providing data with increased external validity. In this sense, the observation of positive results in only 62.5% of the trials (reduced to 50% if considered only between-group analysis) is a fact that should be observed carefully. There is no obvious reason that distinguishes randomized trials that demonstrate significant effects from those that do not, among the studies that composed our sample. However, as a positive consideration, the studies that presented the most appropriate and detailed protocols and analysis were concordant in showing positive effects. Both Borud et al. [34, 40] (a multicentric trial with an up to 12-month follow-up and with possibility of individualized treatment customized to each patient—highly valuable to TCM) and Hachul et al. [35] (the only trial to use polysomnography, the gold standard method for sleep evaluation) reported improvement in sleep-related variables after acupuncture.

### 3.6. Risk of Bias Assessment

The evaluation of risk of bias considered eight articles due to the exclusion of three case reports [29, 37, 38] and a follow-up article by Borud et al. [34]. Additionally, as there was one cohort among the selected articles, the items related to randomization, allocation concealment, and blinding were evaluated only in seven articles.

In general, there was a low risk of bias among the evaluated items. The only item that presented a potential high risk of bias was “blinding of participants and personnel” (28.6%). This is a consequence of the presence of some articles that did not use any kind of placebo or sham group, but rather employed waiting list or no treatment groups as controls. In these cases, as only one group has received acupuncture, it would be impossible to make the participants blinded to the treatment. Other than that, no item displayed concerning increased levels of biases. An overview about the risk of bias in our sample can be found in Table 2.

### 3.7. Evaluation of Articles According to the STRICTA Reporting Guidelines

As with the risk of bias assessment, the follow-up by Borud et al. [34] was excluded from this evaluation, making this analysis with a total of 11 articles. Additionally, subitems 1c, 6a, and 6b were not evaluated in the three case reports, as it can be applied only to comparative studies. Thus, in these subitems, only eight articles were considered. An overview about evaluation of the articles according to the STRICTA guidelines can be found in Table 3.

STRICTA guidelines work as checklist of items that should preferably be reported in all clinical acupuncture trials, rather than as a methodological quality evaluation tool. Thus, more than evaluating the techniques and methods employed, this tool has helped us to evaluate the proportion of articles that have properly addressed these items. In this sense, several items had a good reporting rate, ranging from 80% to 100%, mainly on items related to acupuncture rationale (item 1), treatment regimen (item 3), and control intervention. Conversely, an increased omission rate (i.e., “no” responses) was observed in several items, but mainly on those related to needling details and acupuncturists background, such as in “depth of insertion” (2c; 81.2%), “response sought” (2d; 63.6%), “needle stimulation” (2e; 54.5%), “needle retention time” (2f; 54.5%), “needle type” (2g; 54.5%), and “description of acupuncturists” (5a; 54.5%). When evaluating reporting and omissions rates individually for each article, results are satisfactory: articles presented an average reporting rate of 62.1%, while the average omission rate was of 35.8%. The trial that presented the highest reporting rate (82.4%) and consequently the lowest omission rate (11.8%) was Avis et al. [32]. On the other hand, the lowest reporting rate (42.9%) and highest omission rate (57.1%) were observed in Hu [37]. Curiously, only one among the selected articles (Borud et al. [40]) has cited and referenced STRICTA guidelines in their Methods section.

A full description of how each selected article addressed the items of STRICTA checklist can be checked in Table 3. A summarized evaluation of STRICTA checklist, containing responses and omissions rates per item and per article can be found in Table 4.

## 4. Discussion

The current article presents a systematic literature review about the therapeutic effects of acupuncture on sleep-related complaints in postmenopausal women. Here we highlight one of the biggest challenges of the current review: to join the need of synthesis of general evidences by systematic methods, which is paramount in western medicine, with the patient-focused and customized treatment professed by acupuncture and TCM in general. This discrepancy between western and eastern medicines becomes clear in the way each of them approach sleep complaints observed in postmenopausal women. In western medicine, insomnia and other sleep disorders and complaints may be consequences or symptoms of the postmenopausal period, which are primarily caused by a hormonal imbalance. In this case, the focus of treatment is on the symptoms (as the sleep complaints, aside from hot flushes and behavioral consequences). On the other hand, according to the perspective of TCM, the postmenopausal period can be classified as a syndrome, in which sleep disorder may or may not be present, being explained as a consequence of an energetic imbalance. More specifically, in postmenopause there is a marked deficiency of* yin*, leading to blood and qi stagnation and compromising the balance of kidney, heart, and liver [38, 39]. In this case, more than focusing the treatment on the symptoms, TCM prioritizes the treatment of the general root cause (in this case, the postmenopausal syndrome), as it is understood that by treating this root cause, all the consequent symptoms should improve. One should bear in mind that, despite the difficulty in joining these two contexts, they are both valid, are reasonable in their own theoretical background, and can coexist.

An important tool in the standardization of acupuncture trial was the development of STRICTA, which in some extent is an attempt to synchronize both eastern and western scientific methods. This tool was first published in 2001 [41], but has gained importance upon the publication of its updated version in 2010 [28], being now considered a methodological landmark on acupuncture research. However, as can be seen in selected articles, it looks like that authors are not aware of this checklist, or at least are systematically omitting its use on their articles, as only one record has properly mentioned and cited STRICTA [40]. Of note, most of the selected articles were published after 2001, so the lack of reference to STRICTA in this article is not a temporal problem, as they could have mentioned the previous version of this checklist. Regardless of mentioning STRICTA, it seems that the authors are aware of which are the important items to be reported on an acupuncture trial, as the compliance with the STRICTA reporting items was of 62.1% among the selected articles. So, one may conclude that, despite the fact that the authors (or in a bigger extent, the community involved in acupuncture research) are not fully aware of STRICTA, the importance of reporting these items is commonly understood. An important step to work on is to advertise and propagate STRICTA more prominently, in order to increase awareness about this tool, making it a common reporting guideline among acupuncture trials (as CONSORT is for general randomized clinical trials and PRISMA is for systematic reviews and meta-analyses).

Despite the satisfactory compliance rate with STRICTA items, the selected articles present a high and marked level of heterogeneity, mainly in what regards acupuncture related variables (type of intervention, type of control group, needling procedures, etc.), which reinforces the patient-focused principles of acupuncture. Such a conclusion sets high heterogeneity as a virtually conditional feature of acupuncture trials. Moreover, some degree of variation was also observed on outcome-related variables, such as on time between intervention and outcome measurement and type of evaluation of sleep-related features. Information about the heterogeneity on these items can be checked in Tables 1 and 3. In addition to this increased heterogeneity, it could be observed that there are a limited number of clinical trials specifically devoted to this relationship. Moreover, even among this limited number of articles, sleep is often considered as a secondary endpoint, being the evaluation of hot flushes and vasomotor symptoms the most common primary outcome to be assessed. As a consequence of this limited number of studies directly related to the evaluation of sleep disorders and complaints during the postmenopausal period, as well as to the high heterogeneity among the articles, it becomes challenging to summarize the literature and to compile evidences. The reduced number of articles and increased heterogeneity hampers the possibility of a meta-analysis specifically dedicated to the effects of acupuncture for postmenopausal sleep complaints.

In a wider perspective, Attarian et al. [18] recently performed a systematic review about the available treatments for chronic insomnia in menopause. The authors state that acupuncture cannot be recommended as a therapeutic alternative, mainly due to the low level of evidence among these studies. In agreement with Attarian et al., we further highlight that acupuncture still cannot be definitely indicated as a treatment for postmenopausal sleep problems, not due to its lack of efficacy, but rather due to limited amount of studies conducted in this field. This seems not to be a specific problem of the relationship addressed in the current review, but rather of acupuncture research in general. Meta-analysis on the effects of acupuncture for other clinical conditions has also described a difficulty in obtaining controlled, randomized, and properly blinded studies, which would allow for better meta-analysis and better evidences [19–21]. Thus, facing the aforementioned methodological problems, researchers on acupuncture should be encouraged to perform more replicable studies, and to engage themselves in further randomized and duly controlled trials (following STRICTA guidelines), in order to ultimately provide reliable data on the efficacy and usefulness of this technique.

Some more specific methodological issues deserve special attention, as they lead to additional heterogeneity and cannot be easily overcome. One of them regards the proper development of a sham group with no therapeutic effect [42]. As according to the TCM the principle of acupuncture relies on the balance of energy (named* Chi* or* Qi*), which flows through specific channels on the body, named meridians, it is virtually impossible to consider that there are inert points in the body, which would prevent any attempt of a proper sham group. In spite of that, efforts are made to standardize sham acupoints that have not the same effects as the points that are the focus of each research protocol. In our sample, four out of seven controlled trials have employed sham groups; the remaining used either no-intervention control groups or standard/general acupuncture control groups (Table 2). Another important point relates to the fact that acupuncture was developed based on an energetic diagnosis, which is individual for each patient. As an example, according to the TCM, insomnia may be produced by a broad range of causes and imbalances, thus being possible to be treated by many distinct interventions in different patients. In the current literature, there are different approaches about application of acupuncture in clinical trials. There are those studies that prioritize an energetic diagnosis, providing each patient with an individualized and customized treatment [34, 40], which seems more reasonable under the perspective of TCM. There are others that prioritize the standardization of a selection of acupoints to be equally applied to all patients within a research sample [30], which seems more complete and logical if considered the scientific methodological rigor. Both approaches are reasonable and have their own benefits and caveats, and it is challenging to join both perspectives together in a single trial. Among our sample, only one out of eight comparative trials has employed fully individualized treatments, while four have used identical treatments for all participants. The remaining have used a partially individualized treatment, in which there was a set of common acupoints to be used in all participants, but with the possibility of additional acupoints depending upon individual diagnosis. (Table 3). The background of the acupuncture intervention may also be an important source of variation among trials, as it leads to completely different approaches and therapeutic choices. In this regard, the type and nature of treatment chosen in a given trial (TCM, Japanese, Korean, Western, ear acupuncture, etc.) should always be taken into account. However, for this specific issue, this seems not to be a problem, as most of the articles have employed TCM-based methods. Other important methodological topics possibly leading to variation in results relate to issues such as the personal experience of each acupuncturist and the mode and depth of needle insertion, among other factors [43].

Considering the selected articles, it can be noted that the majority of them are directed only to the approach and treatment of vasomotor symptoms caused by hormonal decay in this life period, being the sleep-related complaints often neglected or considered as a secondary measure. In general, insomnia is strongly associated with the severity and quantity of hot flushes [31], reason why this is the most commonly discussed sleep disorder in this case. However, in a previous Brazilian sample an important prevalence not only of insomnia, but also of OSAS, was observed in postmenopausal women. This is a clinically relevant data, mostly because insomnia complaints are often a misunderstood and misleading symptom. Many times when a patient complains about insomnia, they may be referring to other sleep disorders that lead to similar daytime effects, such as OSAS or periodic limb movements disorder [44]. An incomplete understanding of the current clinical picture may lead to an inadequate treatment and consequent worsening of the previous condition. As an example, the use of benzodiazepines is common for the treatment of insomnia. However, if not adequately prescribed in cases of sleep apnea it can lead to worsening of symptoms, mainly because of the effects of these drugs as muscle relaxants, contributing to the upper airway obstruction [45]. The current review showed a complete absence of articles intending to use acupuncture for sleep disorders other than insomnia in postmenopausal women. It should be noted that previous reports have already demonstrated the efficacy of acupuncture for the treatment of apnea in nonmenopausal samples [22, 23]. Thus, it is reasonable to believe that the same may be observed in women after the menopause.

To a proper understanding of the above presented results and discussion, some limitations and methodological caveats should be taken into consideration. The most evident of them have already been presented, which are the increased heterogeneity and limited number of articles. These both conditions are indeed important limitations and the most important reason to invest in new and better-designed acupuncture trials in the future, thus allowing better conclusions based on bigger and more homogeneous data samples. Another class of previously discussed methodological caveats is the acupuncture-related limitations, among which we highlight the potential bias of sham control groups in acupuncture trials and the variability among interventions. Additionally, some other methodological drawbacks should be discussed. Firstly, one may wonder that in a bibliographic search of articles about acupuncture, a higher proportion of articles in Chinese, or eventually in other western languages such as Japanese or Korean, would be expected. However, only three articles in Chinese were present among the 89 included in our primary samples. From these, one was excluded due to incompatible design at the first step of selection process, while the other two were excluded because full texts were not available, leading us to a final sample composed only by articles in eastern languages (11 in English, one in Spanish). It is highly possible that articles in Chinese and other languages should have been published, but it seems that they are being published in nonindexed or nonsearchable sources, which suggests a case of publication bias. Another important point relates to the fact that only two databases were used for bibliographic search. In this case, further reviews on the topic should include more databases. However, more than including traditional global databases (such as the employed PubMed/Medline and Scopus, as well as Embase and Web of Science), local and specific databases should also be considered, in order to include a bigger amount of articles in general, specifically those in western languages.

## 5. Conclusions

A primary conclusion regards the limited number of articles included. Few studies have addressed the therapeutic effects of acupuncture on sleep-related complaints in postmenopausal women, most of them with reduced sample size. In this sense, it is surprising how this millenary technique has been the focus of scientific reports only in the last few years. Additionally, heterogeneity was an important finding of the current review, both in what regards acupuncture intervention and studies design, which have varied since case reports until multicentric randomized controlled trials. Case reports on the field describe interesting data and controlled clinical trials address the possible effects in larger samples. Controlled trials usually corroborated these effects, but some studies have failed to address beneficial effects of acupuncture. In spite of that, data are still not sufficient to allow concluding that acupuncture is a reliable therapeutic alternative for the treatment of postmenopausal sleep disorders. However, this observation is not due to the lack of efficacy, but rather due to the limited evidences raised so far. Previous results are promising and the tendency is that, as new randomized controlled trials on the field are published, acupuncture can be suggested as a reliable treatment to sleep complaints in postmenopausal women. New researches, duly controlled and scientifically sound, should be encouraged. Additionally, studies not only on general sleep complaints, but also on more specific diseases, such as in insomnia and OSAS, should be performed. Regarding acupuncture intervention, both studies applying fully individualized interventions and standardized treatments are welcome, as both serve for acquisition of evidences, considering that the acupuncture protocol is fully described and replicable. Additionally, as most of the current studies have been performed according to the TCM, other traditional acupuncture techniques (such as Japanese, Korean, Western, French ear acupuncture, etc.) should be encouraged. Finally, the use of STRICTA guidelines is highly recommended for future studies.

Regardless of the still limited evidence about the effects of acupuncture for postmenopausal sleep disorders, none of the selected articles described side or deleterious effects. Thus, acupuncture can be safely applied in clinical practice for this population as an alternative treatment, but not as primary or single conduct.

## Figures and Tables

**Table 1:** List and description of selected studies.

Article	Article type	Sample description	*n*-exp	*n*-ctrl/sham	Treatment condition	Type of treatment	Acupoints, exp	Acupoints, ctrl	Sleep assessment	Main results
Hammes et al., 2014 [38]	Case report	49-y postmenopausal woman	1	NA	22 sessions, weekly or biweekly	Body and auricular acupuncture	Sessions 1–6: LI-4, LR-3, CV-6, CV-17, HT-6, KI-6, KI-2, GV-20, auricular: ShenMen, kidney Sessions 7–22: LI-4, LR-3, CV-4, CV-6, CV-17, HT-6, KI-6, GB-41, GV-20, auricular: ShenMen, kidney	NA	Insomnia self-report	Improvement

Baccetti et al., 2014 [39]	Randomized controlled trial	Postmenopausal women, ≥3 hot flushes episodes/day	47	49	Twice a week, during four weeks	B ody acupuncture	Stimulation with star needles in dorsal region C7-T5. Electrostimulation: dispersion (100 cycles/s) on GV-23, CV-22, BL-2, LI-11, and LI-4; tonification (40 cycles/s) on SP-10 and SP-6; and tonification with GV-20, CV-4, CV-6, ST-37, and LR-3	None	1–4 scale about sleep disorders severity	Improvement

Hachul et al., 2013 [35]	Randomized controlled trial	Postmenopausal women, insomnia	9	9	Twice a week, during five weeks	Body acupuncture	Not reported	Sham acupoints	PSQI and PSG	Improvements in SWS at PSG and in PSQI score

Painovich et al., 2012 [30]	Randomized controlled trial	Peri- and postmenopausal women, at least seven VMS/day	12	9/12	Three times a week, during 12 weeks	Body acupuncture	GV-20, PC-6, HT-7, LV-3, LI-4, LI-11, KI-3, SP-6, ST-36, CV-17, CV-6, GV-14, GB-15, GB-18, GB-20, GB-23, GB-34, and KI-3	Sham or none	PSQI	Nonsignificant

Kung et al., 2011 [36]	Cohort	Postmenopausal women, insomnia	45	NA	Daily before sleep, during four weeks	Auricular acupuncture	ShenMen, kidney, heart, brainstem, and subcortex	NA	PSQI	Improvements in PSQI score, sleep latency, total sleep duration, and sleep efficiency

Borud et al., 2009 [40]	Multicentric randomized controlled trial	Postmenopausal women, at least seven hot flushes/day	134	133	10 sessions over 12 weeks	Body acupuncture (elective additional moxibustion)	Individualized and customized treatment	None	Sleep logs and WHQ	Improvement in total sleep time

Borud et al., 2010 [34]	Follow-up on Borud et al., 2009 [40]	Postmenopausal women, at least seven hot flushes/day	134	133	NA	NA	NA	NA	WHQ	Maintenance of results, as in Borud et al., 2009 [40]

Avis et al., 2008 [32]	Randomized controlled trial	Peri- or postmenopausal women, at least four hot flushes/day	19	18	Twice a week, during eight weeks	Body acupuncture	CV-4, KI-3, SP-6, BL-23, HT-6, KI-7 (possible additional points depending on diagnostic: KI-6, KI-10, GV-4, CV-6, BL-52, LR-3, LR-8, GB-20, BL-18, PC-7, GB-13, GV-20, taiyang, HT-7, HT-8, Yintang, CV-15, BL-15)	Sham acupoints	Women's Health Initiative Insomnia Rating Scale	Nonsignificant

Huang et al., 2006 [31]	Randomized controlled trial	Postmenopausal women, at least 7 moderate or severe hot flushes/day	12	17	Nine sessions, during seven weeks	Body acupuncture	5–7 acupoints from a previously defined list	Sham acupoints	PSQI	Nonsignificant

Hu, 2005 [37]	Case report	51-y postmenopausal woman	1	NA	Three times a week, duration not clear	Body and auricular acupuncture	BL-23, BL-15, PC-7, HT-7, Anmian (extra), SP-6, LR-3, KI-3, BL-18, BL-20, GV-20, GV-24, EX-HN 3 Auricular: subcortex, sympathesis, ear-ShenMen, endocrine, ovary, kidney, spleen, and heart	NA	Insomnia self-report	Improvement

Cohen et al., 2003 [33]	Randomized controlled trial	Postmenopausal woman, self- identification of menopausal hot flashes	8	9	Irregular schedule, total six sessions	Body acupuncture	BL-15, BL-23, BL-32, GV-20, HT-7, PC-6, SP-6, SP-9, LI-3	Standard treatment, not related to menopause	0–3 scale about sleep disorders severity	Nonsignificant between groups comparison Improvement in within-group comparison

Bijak, 2009 [29]	Case report	59-y postmenopausal woman	1	NA	Once a week, ten weeks	Body and auricular acupuncture	BL-17 (until the 4th session), BL-43 (5th until the 10th session), KI-3, BL-31, HT-3, LI-3, auricular: Jerome's point and sleep point (until the 4th session)	NA	Insomnia self-report	Improvement

Exp: experimental group; ctrl: control group; *n*-exp: sample size at experimental group; *n*-ctrl: sample size at control group; NA: not applicable; PSQI: Pittsburgh Sleep Quality Index; PSG: Polysomnography; WHQ: Women's Health Questionnaire; SWS: slow wave sleep.

**Table 2:** Risk of bias assessment.

	Low risk	Unclear risk	High risk
Sequence generation (*n* = 7)	100.0%	0.0%	0.0%
Allocation concealment (*n* = 7)	85.7%	14.3%	0.0%
Blinding of participants and personnel (*n* = 7)	71.4%	0.0%	28.6%
Blinding of outcome assessors (*n* = 7)	57.1%	42.9%	0.0%
Incomplete outcome data (*n* = 8)	100.0%	0.0%	0.0%
Selective outcome reporting (*n* = 8)	100.0%	0.0%	0.0%
Other sources of bias (*n* = 8)	100.0%	0.0%	0.0%

Evaluation according to the Cochrane risk of bias tool.

**Table 3:** Evaluation of the selected articles based on STRICTA criteria.

		Hammes et al., 2014 [38]	Baccetti et al., 2014 [39]	Hachul et al., 2013 [35]	Painovich et al., 2012 [30]	Kung et al., 2011 [36]	Borud et al., 2009 [40]	Avis et al., 2008 [32]	Huang et al., 2006 [31]	Hu, 2005 [37]	Cohen et al., 2003 [33]	Bijak, 2009 [29]
(1) Acupuncture rationale	(1a) Style of acupuncture	Yes, TCM	Yes, TCM, five elements	Yes, TCM	Yes, TCM	Yes, ear acupuncture	Yes, TCM	Yes, TCM	Yes, TCM	Yes, TCM, ear acupuncture	Yes, TCM	Yes,TCM, ear acupuncture
	(1b) Reasoning for treatment provided	Yes	Yes	No	Yes	Yes	Yes	Yes	Yes	Yes	Yes	Yes
	(1c) Extent to which treatment was varied	N/A, case report	Yes, partially individualized	Yes, no individualization	Yes, no individualization	Yes, no individualization	Yes, individualized treatment	Yes, partially individualized	Yes, partially individualized	N/A, case report	Yes, No individualization	N/A, case report

(2) Detail of needling	(2a) Number of needle insertions per subject per session	No	Yes for common protocol; No for individualized treatment	No	Yes, 28 points	Yes, 5 auricular points	No	Yes, 11 to 16 needle insertions per subject per section	Yes, 5–7 points	Yes, 21 points + 9 ear points	Yes, 9 points	Yes, 5 points + 2 ear points
	(2b) Names or location of points used	Yes^*∗*^	Partially, Yes^*∗*^ for common protocol; No for individualized treatment	No	Yes^*∗*^	Yes^*∗*^	No	Partially, Yes^*∗*^ for common protocol; No for individualized treatment	Yes^*∗*^	Yes^*∗*^	Yes^*∗*^	Yes^*∗*^
	(2c) Depth of insertion	No	No	No	Yes, 0.5 to 1.5 in	No	No	Yes, 0.5 to 3 cm	No	No	No	No
	(2d) Response sought	Yes, needles inserted until deqi sensation was obtained	No	No	Yes, manually stimulated until reaching *deqi*	No	Yes, deqi obtained, if possible	Yes, deqi obtained, if possible	No	No	No	No
	(2e) Needle stimulation	Yes, needling with tonifying technique	Yes^*∗*^	No	Yes, manually stimulated until reaching *deqi*	No	Partially, needle manipulation allowed	No	No	No	No	Yes, superficial tonification
	(2f) Needle retention time	Yes, 20 min	Yes, 30 min	Yes, 30 min	Yes, 30 min	No	No	Yes, 20 min for anterior points; 10 min for posterior points	No	No	No	No
	(2g) Needle type	Yes, DBC Spring-Ten sterile and disposable needles 0.20 × 0.30 mm	No	Yes, disposable 0.25 × 40 mm acupuncture needles	No	Yes, 0.2 cm sized magnetic pellet on a 1 cm sized sticky patch (Ching-Ming Co., Taiwan)	No	Yes, Sterile, single-use, Vinco 34-gauge, 1-inch (0.22 25 mm) and 30-gauge, 1.5-inch (0.30 40 mm)	No	No	No	Yes, disposable 0.3 mm needles and ASP needles for auriculotherapy

(3) Treatment regimen	(3a) Number of treatment sessions	Yes^*∗*^	Yes^*∗*^	Yes^*∗*^	Yes^*∗*^	No	Yes^*∗*^	Yes^*∗*^	Yes^*∗*^	No	Yes^*∗*^	Yes^*∗*^
	(3b) Frequency and duration of treatment sessions	Yes^*∗*^	Yes^*∗*^; ca. 50 min/session	Yes^*∗*^	Yes^*∗*^	Yes^*∗*^	Yes^*∗*^	Yes^*∗*^	Yes^*∗*^; 20 min per section	Yes^*∗*^	Yes^*∗*^	Yes^*∗*^

(4) Other components of treatment	(4a) Details of other interventions administered to the acupuncture group	Yes, description about medicines	Yes, diet (according to TCM) and self-massage (TuiNa)	No	No	No	Yes, moxibustion was allowed	No	No	No	No	No
	(4b) Setting and context of treatment, including instructions to practitioners, and information and explanations to patients	No	Yes, participants received instructions about diet and self-massage Practitioners received specific training before the beginning of the study	No	No	No	Yes, participants free to use self-provided care. Acupuncturists discussed the expected TCM diagnoses and the recommended acupuncture point selection	Yes, all study participants were instructed not to take hormonal medications or initiate other treatments for their hot flashes during treatment	Yes, acupuncturists were instructed to limit verbal contact to the most pertinent clinical information and to refrain from counseling and offering advice	Yes, take light food, more fruits and vegetables, and avoid greasy and spicy food, do appropriate physical exercises to strengthen the body constitution (among others)	No	No

(5) Practitioner background	(5a) Description of participating acupuncturists	No	Yes, sessions were conducted by MDs trained at the TCM with at least 400 h of experience Practitioners received training and were supervised by the director of the study	No	No	Yes, licensed acupuncturist	Yes, the study acupuncturists met the current membership criteria of the Norwegian Acupuncture Society (2,500 h) and had at least 3 years of experience	Yes, treatments were performed by experienced acupuncturists trained in TCM	No	No	Yes, licensed acupuncturist	No

(6) Control or comparator interventions	(6a) Rationale for the control or comparator in the context of the research question	N/A, case report	Yes, control group were given diet and self-massage training, but no acupuncture	Yes, Sham group	Yes, Sham group	No	Yes, control group with no intervention	Yes, Sham group	Yes, Sham group	N/A, case report	Yes, comparison acupuncture treatment	N/A, case report
	(6b) Precise description of the control or comparator	N/A, case report	Yes, TuiNa massage (30 min massage for the areas in which acupuncture points are present) and diet (food choice based on the energetic balance of the individual)	Yes, needles in different acupoints	Yes, Sham points were proximate to the treatment points site, but not considered active	No	Yes, no medical treatment, but free to use self-provided nonpharmaceutical interventions	Yes, needling at sites thought to have minimal effects on hot flashes. Six needles in each side, shallowly needled, no attempt to reach deqi	Yes, Streitberger placebo needles in 4-5 points	N/A, case report	Yes, general treatment: LV4, KI7, ShenMen sympathetic, kidney, liver, and lung points	N/A, case report

STRICTA: Standards for Reporting Interventions in Clinical Trials of Acupuncture; N/A: nonapplicable; ^*∗*^data detailed in Table 1.

**Table 4:** Simplified evaluation according to STRICTA criteria and reporting and omission rates.

		Hammes et al., 2014 [38]	Baccetti et al., 2014 [39]	Hachul et al., 2013 [35]	Painovich et al., 2012 [30]	Kung et al., 2011 [36]	Borud et al., 2009 [40]	Avis et al., 2008 [32]	Huang et al., 2006 [31]	Hu, 2005 [37]	Cohen et al., 2003 [33]	Bijak, 2009 [29]	Reporting rate per subitem	Omission rate per subitem
(1) Acupuncture rationale	(1a) Style of acupuncture	Yes	Yes	Yes	Yes	Yes	Yes	Yes	Yes	Yes	Yes	Yes	100.0%	0.0%
	(1b) Reasoning for treatment provided	Yes	Yes	No	Yes	Yes	Yes	Yes	Yes	Yes	Yes	Yes	90.9%	9.1%
	(1c) Extent to which treatment was varied	N/A	Yes	Yes	Yes	Yes	Yes	Yes	Yes	N/A	Yes	N/A	100.0%	0.0%

(2) Detail of needling	(2a) Number of needle insertions per subject per session	No	Yes	No	Yes	Yes	No	Yes	Yes	Yes	Yes	Yes	72.7%	27.3%
	(2b) Names or location of points used	Yes	Partially	No	Yes	Yes	No	Partially	Yes	Yes	Yes	Yes	63.6%	18.2%
	(2c) Depth of insertion	No	No	No	Yes	No	No	Yes	No	No	No	No	18.2%	81.8%
	(2d) Response sought	Yes	No	No	Yes	No	Yes	Yes	No	No	No	No	36.4%	63.6%
	(2e) Needle stimulation	Yes	Yes	No	Yes	No	Partially	No	No	No	No	Yes	36.4%	54.5%
	(2f) Needle retention time	Yes	Yes	Yes	Yes	No	No	Yes	No	No	No	No	45.5%	45.5%
	(2g) Needle type	Yes	No	Yes	No	Yes	No	Yes	No	No	No	Yes	45.5%	54.5%

(3) Treatment regimen	(3a) Number of treatment sessions	Yes	Yes	Yes	Yes	No	Yes	Yes	Yes	No	Yes	Yes	81.8%	18.2%
	(3b) Frequency and duration of treatment sessions	Yes	Yes	Yes	Yes	Yes	Yes	Yes	Yes	Yes	Yes	Yes	100.0%	0.0%

(4) Other components of treatment	(4a) Details of other interventions administered to the acupuncture group	Yes	Yes	No	No	No	Yes	No	No	No	No	No	27.3%	72.7%
	(4b) Setting and context of treatment, including instructions to practitioners, and information and explanations to patients	No	Yes	No	No	No	Yes	Yes	Yes	Yes	No	No	45.5%	54.5%

(5) Practitioner background	(5a) Description of participating acupuncturists	No	Yes	No	No	Yes	Yes	Yes	No	No	Yes	No	45.5%	54.5%

(6) Control or comparator interventions	(6a) Rationale for the control or comparator in the context of the research question	N/A	Yes	Yes	Yes	No	Yes	Yes	Yes	N/A	Yes	N/A	87.5%	12.5%
	(6b) Precise description of the control or comparator	N/A	Yes	Yes	Yes	No	Yes	Yes	Yes	N/A	Yes	N/A	87.5%	12.5%

Reporting rate per article		71.4%	76.5%	47.1%	76.5%	47.1%	64.7%	82.4%	58.8%	42.9%	58.8%	57.1%		
Average reporting rate among articles		62.1%												
Omission rate per article		28.6%	17.6%	52.9%	23.5%	52.9%	29.4%	11.8%	41.2%	57.1%	35.3%	42.9%		
Average omission rate among articles		35.8%												

STRICTA: Standards for Reporting Interventions in Clinical Trials of Acupuncture; N/A: nonapplicable.
